# Supplementary material for: Structural basis of superinfection exclusion by bacteriophage T4 Spackle
Source: Commun Biol. 2020 Nov 19;3:691. doi: 10.1038/s42003-020-01412-3 (PMC7677548; doi:10.1038/s42003-020-01412-3)
Supplement: Supplementary file 1 — Supplementary Information [file 42003_2020_1412_MOESM1_ESM.pdf]

Supplementary Information

Structural basis of superinfection exclusion by bacteriophage T4 Spackle

Ke Shi[1,2], Justin T. Oakland[1,2], Fredy Kurniawan[1,2], Nicholas H. Moeller[1,2], Surajit Banerjee[3], Hideki Aihara[1,2*]

[1] Department of Biochemistry, Molecular Biology and Biophysics, University of Minnesota, 321 Church Street S.E., Minneapolis, MN 55455, USA

[2] Institute for Molecular Virology, University of Minnesota, Minneapolis, MN 55455, USA

[3] Northeastern Collaborative Access Team, Cornell University, Advanced Photon Source, Lemont, IL 60439, USA

[*]Correspondence email: aihar001@umn.edu

This PDF file includes:

Supplementary Figures S1 to S8

Supplementary References

Supplementary Figures

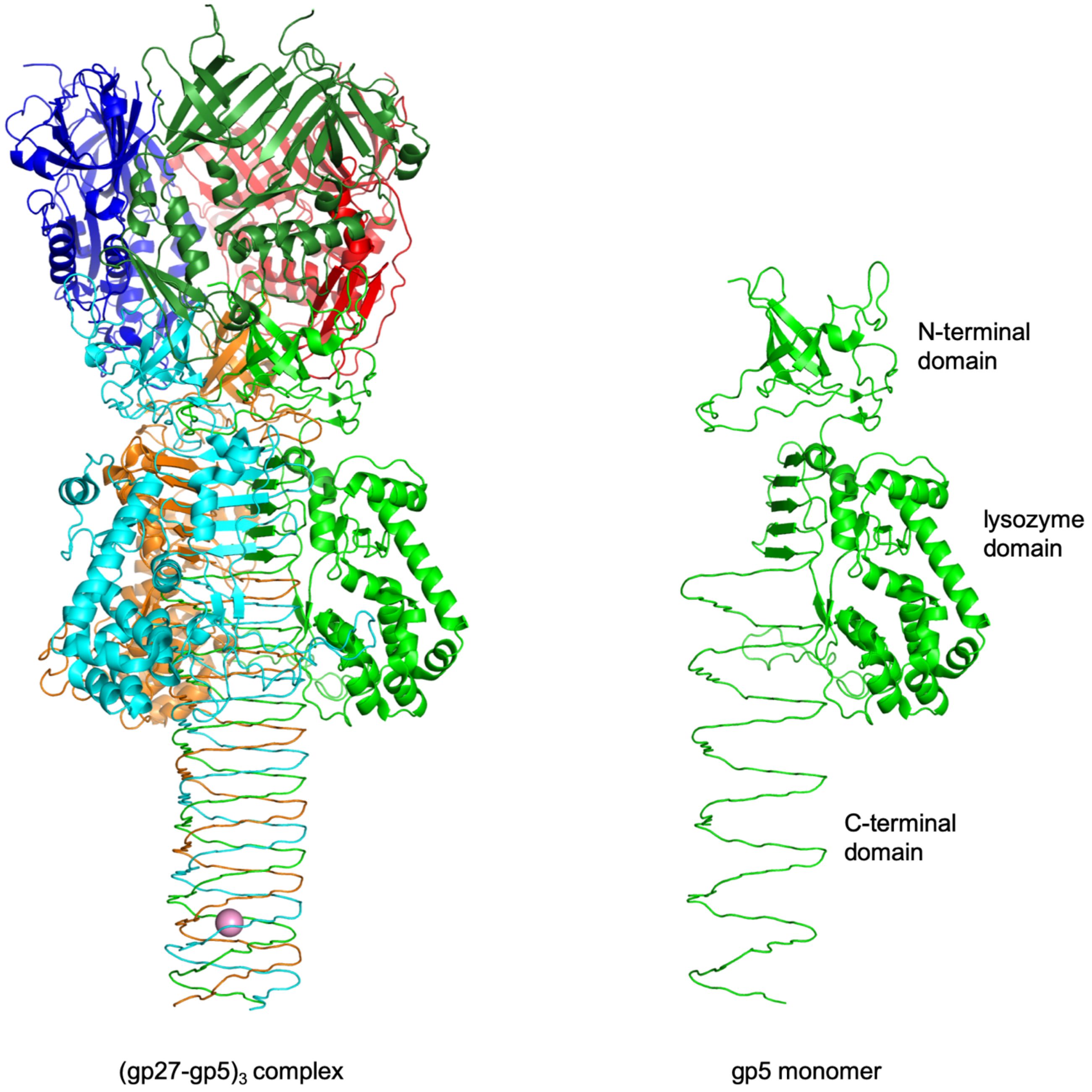

Supplementary Fig. S1 | Structure of the T4 (gp27-gp5)$_3$ complex. Crystal structure of the non-cleavage phenotype mutant of gp5, S351L, complexed with gp27 reported earlier (1wth)[1]. The gp5 monomers are shown in light green, cyan, and orange, whereas the gp27 monomers are in forest green, blue, and red. A gp5 monomer within the complex is shown on the right, with its 3 domains labeled. The pink sphere represents a potassium ion.

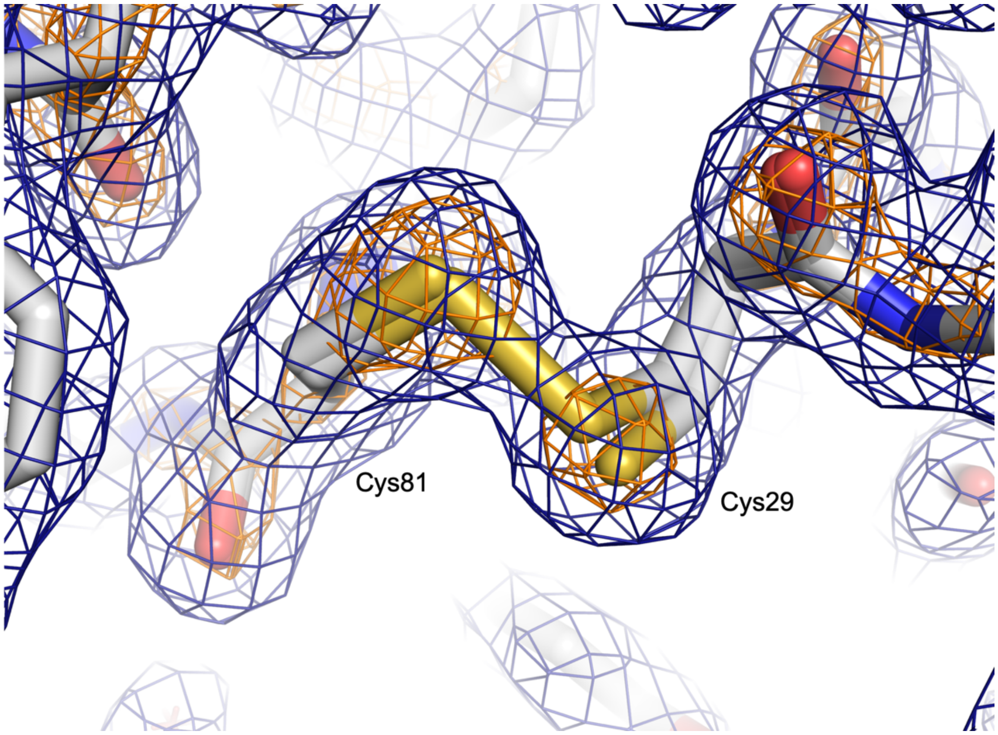

Supplementary Fig. S2 | Partial disulfide linkage. Electron density map for the region surrounding Cys29 and Cys81 of Spackle in complex with gp5 lysozyme. 2mFo-DFc map contoured at 1.0σ (blue mesh) or 3.0σ (orange) are overlaid on the refined protein model shown as sticks.

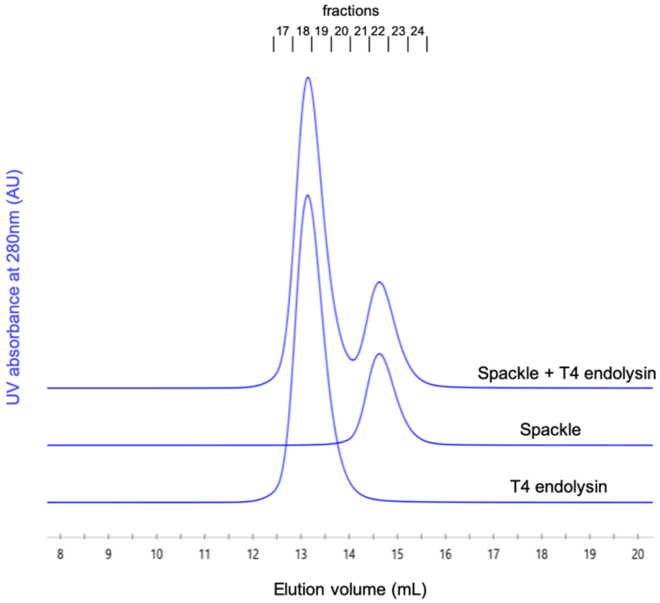

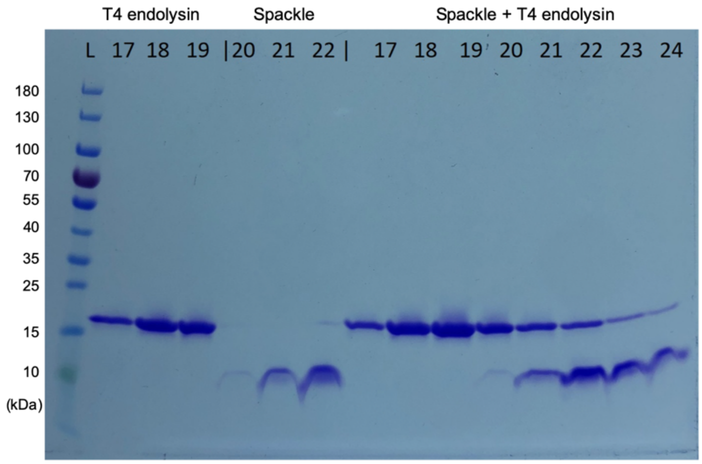

Supplementary Fig. S3 | Spackle does not form a stable complex with T4 endolysin. Left: An overlay of the SEC profiles of 3 separate injections: Spackle alone, T4 endolysin alone, and the mixture between the two. Protein contents of the collected fractions were analyzed by SDS-PAGE (right). L: ladder of protein standards.

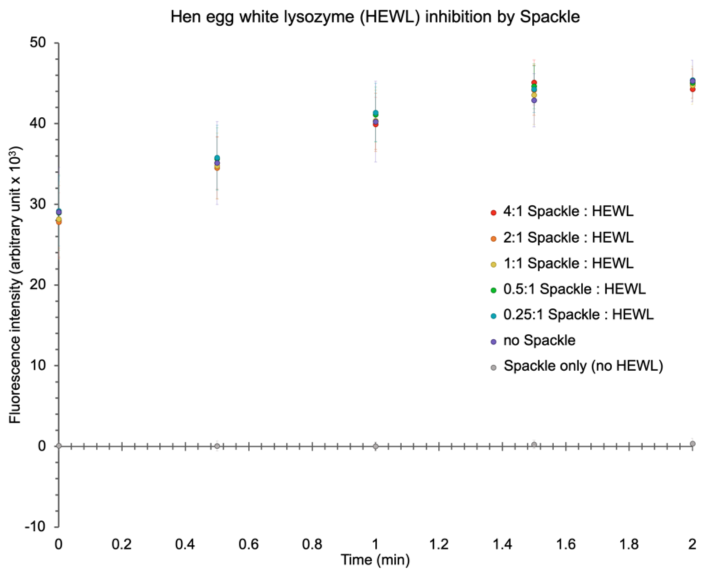

Supplementary Fig. S4 | Spackle does not inhibit hen egg white lysozyme activity. Cell wall degrading activity of HEWL measured in the presence of varying amounts of Spackle. Due to high activity of HEWL, the reaction proceeded significantly before the first time point (0 min) of fluorescence intensity measurement.

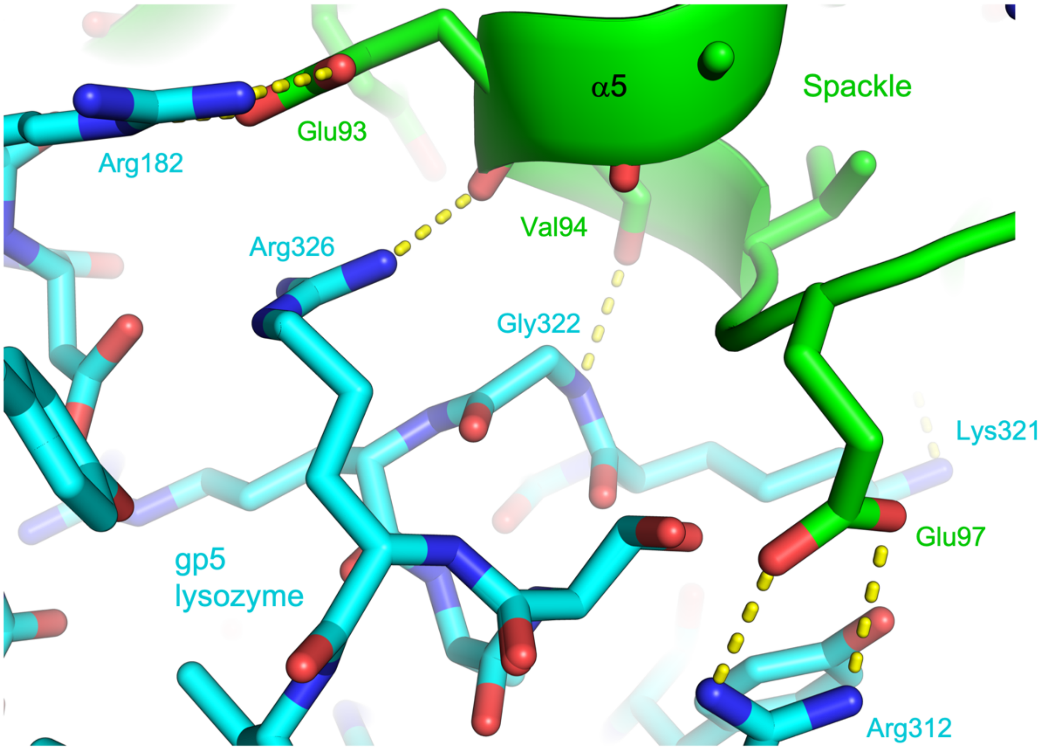

Supplementary Fig. S5 | Zoomed in view of the interface around Gly322 of gp5. Amino acid residues of Spackle (green) and gp5 lysozyme (cyan) involved in direct interaction are labeled. Substitution of Asp or Asn for Gly322 of gp5 (as in 5ts1 mutant and T4 endolysin, respectively) would cause a steric clash with α5 of Spackle.

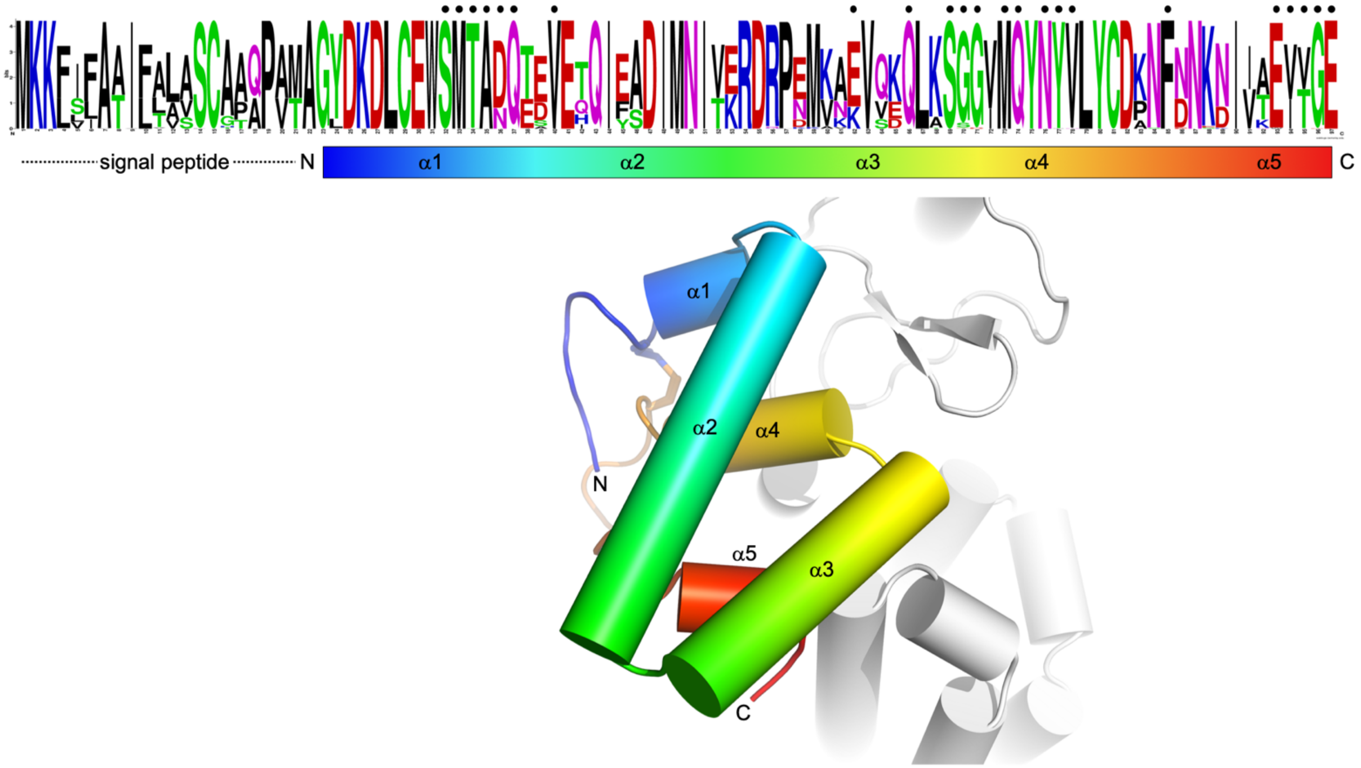

Supplementary Fig. S6 | Sequence conservation among Spackle homologs. Weblogo[2] plot showing the conservation of a total of 178 protein sequences available in the UniProt database that share 50% or higher identity with T4 Spackle (UniRef50_P39230). Residues within 4 Å of gp5 lysozyme are marked by black circles at the top. The structure of Spackle bound to gp5 lysozyme and the rectangle underneath the sequence logo are colored in a gradient of blue to red from the N- to C-terminus (after removal of the signal peptide).

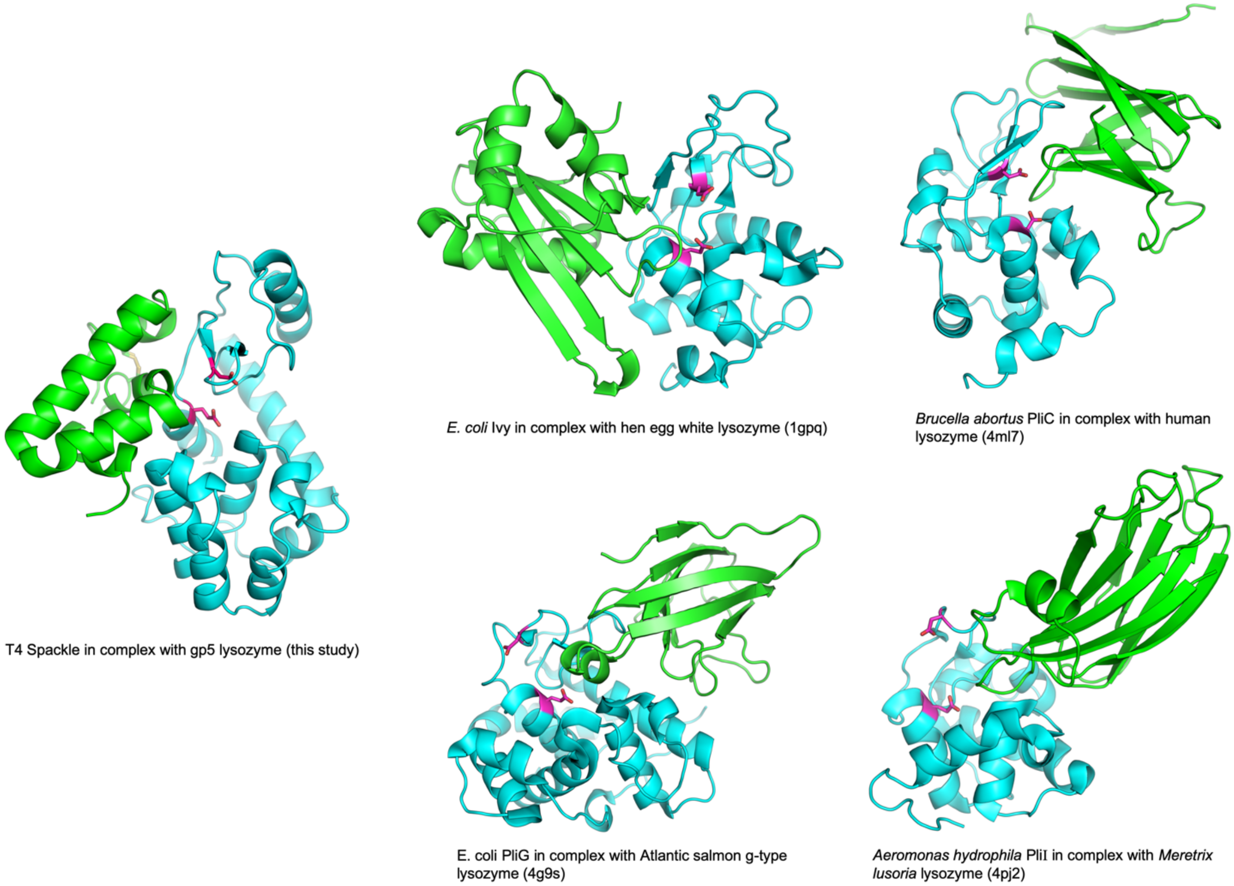

Supplementary Fig. S7 | Structures of various lysozyme-inhibitor complexes. T4 Spackle and the four bacterial lysozyme inhibitors, Ivy[3], PliC[4], PliG[5], and PliI[6] are colored in green. Lysozymes are colored in cyan with the active site residues shown as magenta sticks.

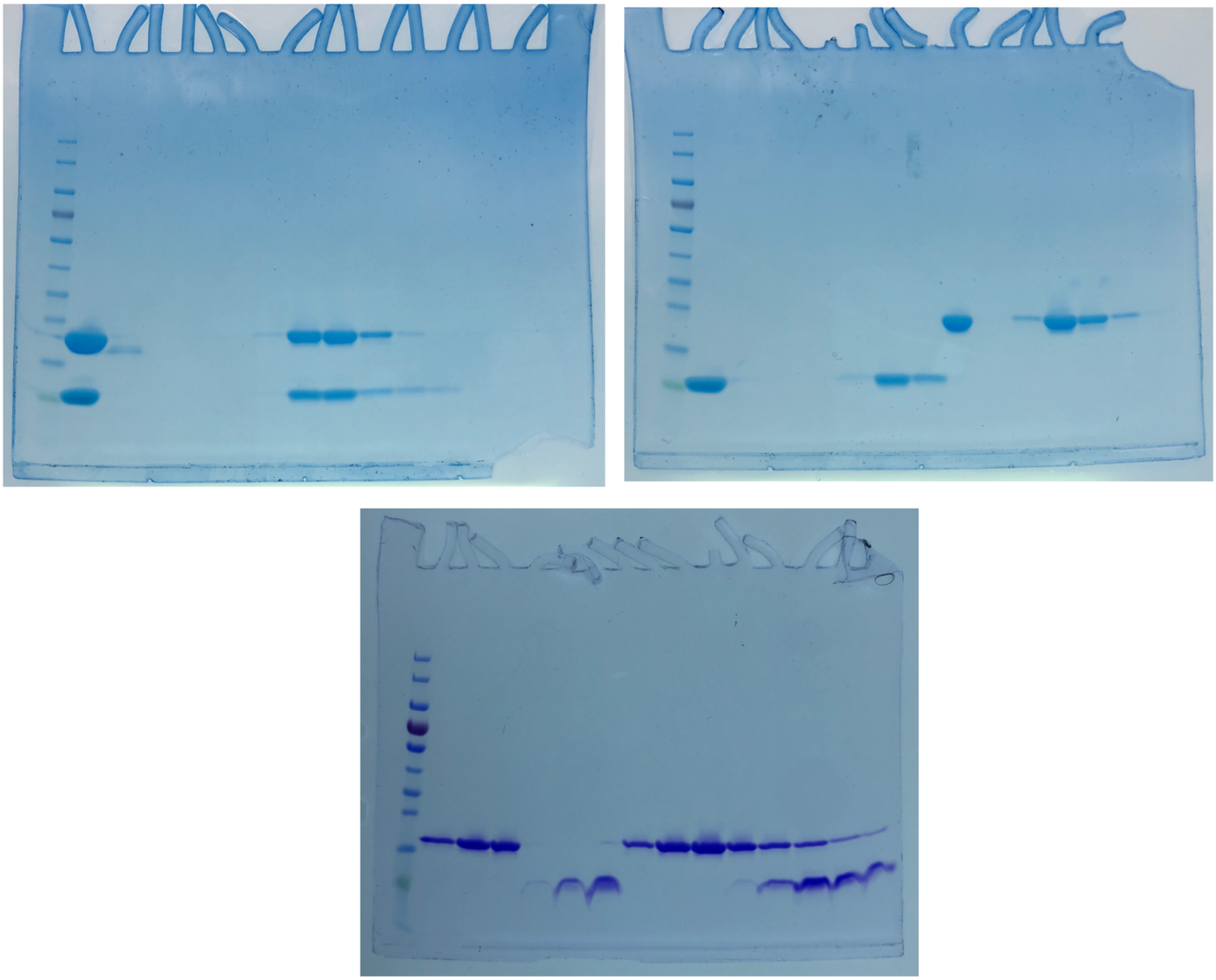

Supplementary Fig. S8 | Uncropped gel images for data shown in **Fig. 1** and **supplementary Fig. S3**

Supplementary References

1. Kanamaru, S., Ishiwata, Y., Suzuki, T., Rossmann, M.G. & Arisaka, F. Control of bacteriophage T4 tail lysozyme activity during the infection process. *J Mol Biol* **346**, 1013-20 (2005).
2. Crooks, G.E., Hon, G., Chandonia, J.M. & Brenner, S.E. WebLogo: a sequence logo generator. *Genome Res* **14**, 1188-90 (2004).
3. Abergel, C. et al. Structure and evolution of the Ivy protein family, unexpected lysozyme inhibitors in Gram-negative bacteria. *Proc Natl Acad Sci U S A* **104**, 6394-9 (2007).
4. Um, S.H. et al. Structural basis for the inhibition of human lysozyme by PliC from Brucella abortus. *Biochemistry* **52**, 9385-93 (2013).
5. Leysen, S., Vanderkelen, L., Weeks, S.D., Michiels, C.W. & Strelkov, S.V. Structural basis of bacterial defense against g-type lysozyme-based innate immunity. *Cell Mol Life Sci* **70**, 1113-22 (2013).
6. Leysen, S. et al. The structure of the proteinaceous inhibitor PliI from Aeromonas hydrophila in complex with its target lysozyme. *Acta Crystallogr D Biol Crystallogr* **71**, 344-51 (2015).
