# Supplementary material for: Structural basis of superinfection exclusion by bacteriophage T4 Spackle
Source: Commun Biol. 2020 Nov 19;3:691. doi: 10.1038/s42003-020-01412-3 (PMC7677548; doi:10.1038/s42003-020-01412-3)
Supplement: Supplementary file 3 — Description of Additional Supplementary Files [file 42003_2020_1412_MOESM3_ESM.pdf]

Description of Additional Supplementary Files

File Name: Supplementary Data 1
Description: **Source data file for Figure 5 and Supplementary Figure S4.** Fluorescence intensity readings (raw data) from the cell wall degradation assay. Individual data points from quadruplicated experiment as well as their average values before and after background subtraction are shown for T4 gp5 lysozyme (worksheet 1-2), T4 endolysin (worksheet 3-4), and hen egg white lysozyme (worksheet 5-6). Cells colored in red represent data points not reliably quantitated due to detector saturation or other technical problems.
